# EFFECTS OF FRAILTY AND FUNCTIONAL IMPAIRMENT ON THE ASSOCIATIONS OF COGNITIVE IMPAIRMENT AND DEMENTIA WITH HEALTHCARE COSTS

**DOI:** 10.1093/geroni/igae098.3314

**Published:** 2024-12-31

**Authors:** Howard Fink, Kerry Sheets, Lisa Langsetmo, John Schousboe, Allyson Kats, Kristine Ensrud

**Affiliations:** Minneapolis Veterans Affairs Health Care System, Minneapolis, Minnesota, United States; Hennepin Healthcare, Minneapolis, Minnesota, United States; Minneapolis Veterans Affairs Health Care System, Minneapolis, Minnesota, United States; HealthPartners, St. Paul, Minnesota, United States; University of Minnesota, Minneapolis, Minnesota, United States; University of Minnesota, Minneapolis, Minnesota, United States

## Abstract

Dementia and cognitive impairment (DCI) are associated with higher total healthcare costs (THC). We sought to determine the fraction of incremental THC of DCI accounted for by frailty and/or functional impairment. We utilized a multi-cohort dataset of 8165 community-dwelling, fee-for-service Medicare beneficiaries (53% women, 79% non-Hispanic White, mean age 79 years) who had index exams in 1 of 4 prospective cohort studies linked with Medicare claims. Cohort-based DCI was defined by self-or-proxy report of a dementia diagnosis or abnormal cognitive test results. Multimorbidity was count of chronic conditions. Frailty was a claims-based deficit accumulation index (CFI) or the Cardiovascular Health Study phenotype (3+ components of shrinking, slowness, weakness, poor energy, low physical activity). Functional impairment was self-reported difficulty performing 4 ADLs (with none, 1, 2, 3 or 4 of walking, climbing steps, transferring, bathing/showering). Annualized THC were ascertained for 36 months following index exams. After adjustment for age, race, geographic region, and multimorbidity, those with cohort-based DCI vs. no DCI had higher incremental THC ($6883 in women and $7276 in men). Respectively, 40%, 31%, 33% and 63% of incremental THC in women and 28%, 29%, 40% and 58% of incremental THC in men were attributable to CFI, phenotypic frailty, functional impairments or a combination of all 3 measures. In conclusion, in both men and women, most of the incremental THC of DCI were attributable to frailty and functional impairment. Further attention should be given to assessment of these geriatric syndromes and potential mitigation of their impact among those with DCI.

